# Sequentially Triggered Nanoparticles with Tumor Penetration and Intelligent Drug Release for Pancreatic Cancer Therapy

**DOI:** 10.1002/advs.201701070

**Published:** 2018-02-26

**Authors:** Xi He, Xinli Chen, Lisha Liu, Yu Zhang, Yifei Lu, Yujie Zhang, Qinjun Chen, Chunhui Ruan, Qin Guo, Chao Li, Tao Sun, Chen Jiang

**Affiliations:** ^1^ Key Laboratory of Smart Drug Delivery Ministry of Education State Key Laboratory of Medical Neurobiology Department of Pharmaceutics School of Pharmacy Fudan University Shanghai 200032 China

**Keywords:** drug delivery, extracellular matrix, pancreatic therapy, redox‐controlled release, tumor penetration

## Abstract

Pancreatic ductal adenocarcinoma (PDAC) is the most aggressive malignancy with a five year survival rate of <5%. The aberrant expression of extracellular matrix (ECM) in the tumor stroma forms a compact physical barrier, which that leads to insufficient extravasation and penetration of nanosized therapies. To overcome the severe resistance of PDAC to conventional therapies, a sequentially triggered nanoparticle (aptamer/cell‐penetrating peptide‐camptothecin prodrug, i.e., Apt/CPP‐CPTD NPs) with tumor penetration and intelligent drug release profile is designed. An ECM component (tenescin‐C) targeting aptamer (GBI‐10) is modified onto stroma‐permeable cell‐penetrating peptide (CPP) for the in vivo CPP camouflage and PDAC‐homing. In PDAC stroma, tenascin‐C can detach GBI‐10 from CPP and exposed CPP can facilitate further PDAC penetration and tumor cell endocytosis. After being endocytosed into PDAC cells, intracellular high redox potential can further trigger controlled chemodrug release. Apt/CPP‐CPTD NPs show both deep penetration in vitro 3D PDAC spheroids and in vivo tumor sections. The relatively mild in vitro cytotoxicity and excellent in vivo antitumor efficacy proves the improved PDAC targeting drug delivery and decreased systemic toxicity. The design of ECM‐redox sequentially triggered stroma permeable NPs may provide a practical approach for deep penetration of PDAC and enhanced drug delivery efficacy.

## 1 Introduction

Pancreatic ductal adenocarcinoma (PDAC) is an aggressive malignancy with a 5‐year survival rate below 5%.^1^ Its aggressive nature resides in the abnormally high expression of extracellular matrix in the tumor stroma, which forms a compact physical barrier for the delivery of cytotoxic chemotherapeutics into the tumor cells.^2^ Nanomedicines such as liposomes, polymeric nanopraticles, and micelles have been developed to optimize the drug delivery of anticancer agents by accumulation in most tumor site via the enhanced permeability and retention (EPR) effect.^3^ However, the abnormally high dense tumor stroma and hypovascularity in PDAC extracellular matrix (ECM), greatly compromising the tumor‐penetrating performance, and leading to the treatment failure for pancreatic cancer treatment.[[qv: 2a,4]] To address these problems, tremendous efforts have been devoted to the development of advanced drug delivery systems, which are capable of orchestrating several specific interactions in a coordinated pattern and successively overcoming the biological barriers to maximize the therapeutic potency.^5^ Recent years, there is an increasing interest in targeting different component of PDAC stroma and several preclinical studies in modulating ECM density for PDAC treatment.^6^ Coadministration of Nab‐paclitaxel and gemcitabine was one of the most famous strategies hypothesized to target the stroma, which specially collapsed the PDAC stroma accompanied by a marked distortion of the collagen and tumor vascularization.[[qv: 6b,c]] However, by removal of stroma in PDAC, there still remains a concern that this therapy method might provide new space for tumor proliferation and increase the probability of metastasis.^7^ Therefore, there is an urgent need to develop novel strategies to improve therapeutics' stroma penetrating ability without damaging the ECM tumor barrier.

Cell‐penetrating peptide (CPP), a short positive synthetic peptide, performs excellent therapeutic agents permeability after intratumoral injection.^8^ CPP shows excellent in vitro cell internalization capability, however, meanwhile undesired tumor accumulation in vivo due to the lack of selectivity for targeting cells.^9^ To avoid the off‐target accumulation and improved penetration of CPP mediated nanomedicine in vivo, we intend to seek a negatively charged camouflage with tumor‐homing property.[[qv: 9a]] Such camouflage can shield CPP via electrostatic attraction to prevent the nonspecific internalization during circulation, and help the nanomedicine accumulate in the pancreatic lesion via the tumor‐homing property in the meantime. The pancreatic cancer stroma is composed of ECM components, which interact closely with pancreatic tumor cells to create a tumor promoting microenvironment for proliferation or metastasis.^10^ Among the ECM components, collagen I and fibronectin are the key components of stroma in both primary and metastatic sites, protecting cancer cells against apoptosis and accelerating cancer growth.^11^ Tenascin‐C, an overexpressed ECM protein, interacts with other ECM components (e.g., fibronectin, collagen I) and cell surface receptors, playing a central role in oncogenesis pathway in PDAC.^12^ GBI‐10 aptamer is a type of ssDNA that is selected by systematic evolution of ligands by exponential enrichment (SELEX) against tenascin‐C,^13^ which shows reduced susceptibility to biodegradation, as well as high affinity and specificity to tenascin‐C. The inherent negative charge of GBI‐10 aptamer could play as a suitable camouflage for CPP and induce nanomedicine accumulation in pancreatic cancer lesions via EPR effect and tenascin‐C targeting ability. Due to the high affinity to tenascin‐C, the interactions between GBI‐10 and tenascin‐C overrule the electrostatic attraction between GBI‐10 and CPP, thus induce the detachment of GBI‐10 and exposure of CPP for the further intratumoral penetration and tumor cell's endocytosis.

Apart from tumor penetration, efficient release of therapeutic agents is another critical exponent in PDAC treatment.^14^ Multiple external triggers such as pH, light, temperature, redox reactions have been used to induce on‐demand drug release.^15^ Among them, the huge redox potential difference between intracellular tumor cells and normal physiological conditions has been widely chosen as an ideal trigger for drug‐release in nanomedicine design. Intracellular redox potential possesses approximately three orders of magnitude higher glutathione level (≈10 × 10^−3^ m) as compared to the extracellular environment (≈10 × 10^−6^ m).^16^ In addition, with the copresence of the specific reducing enzyme, gamma interferon‐inducible lysosomal thiol reductase,^17^ general redox and thiol responsive materials containing disulfide linkage can stay stable at oxidizing extracellular conditions, but undergo a rapid reduction or thiol‐disulfide exchange inside cancer cells.^18^ In view of the above, we constructed a disulfide‐containing dimeric camptothecin prodrug (CPTD) to obtain the redox‐responsive property with a temporally and spatially controlled drug release intracellularly. By encapsulating CPTD into biodegradable amphiphilic polypeptide copolymer, we successfully developed a redox‐responsive nanoparticle with extremely high drug loading rate.

In this work, we reported the design of a sequentially responsive nanoparticle with ECM‐triggered tumor penetration and redox responsive drug release profile. To realize deep tissue penetration and cellular uptake, we encapsulated the CPTD nanopraticles (NPs) with CPP‐modified amphiphilic copolymer. The positively charged CPP could be shielded by tenascin‐C targeting aptamer GBI‐10, to reduce the untargeted systemic accumulation and increase pancreatic lesion accumulation. Based on the original design, NPs could go through the detachment of GBI‐10 and exposure of CPP after the accumulation in PDAC tissue at tenascin‐C‐highly‐expressed tumor microenvironment. And the exposed CPP could further induce the deep penetration and cellular endocytosis of NPs. After internalized into PDAC cells, the disulfide bond in the prodrug could further be cleaved under intracellular high redox potential to subsequently induce the upregulated antitumor activity.

In summary, the novel Apt/CPP‐CPTD NPs were rationally designed with the following features: (i) GBI‐10 aptamer could perform as CPP camouflage and tumor‐homing ligand to enhance nanomedicine tumor accumulation; (ii) EMC‐triggered CPP exposure realized the efficient tumor penetration and tumor cell endocytosis; (iii) disulfide‐containing CPT prodrug showed an on‐demand drug release behavior. The unique sequentially triggered nanoparticle with tumor penetration and intelligent drug release could serve well as a promising strategy for PDAC treatment.

## 2 Results and Discussion

### 2.1 Design and Preparation of Sequentially Triggered Apt/CPP‐CPTD NPs

**Scheme 1:**
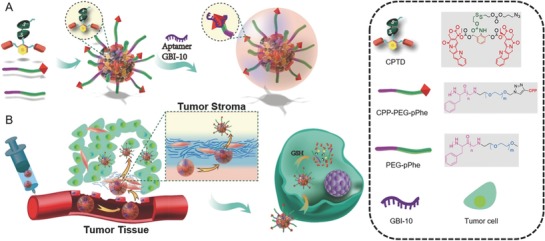
A) Schematic illustration of Apt/CPTD NPs formulation. B) Sequentially triggered tumor stroma permeability of Apt/CPTD NPs and intracellular redox‐triggered drug release.

**Figure 1:**
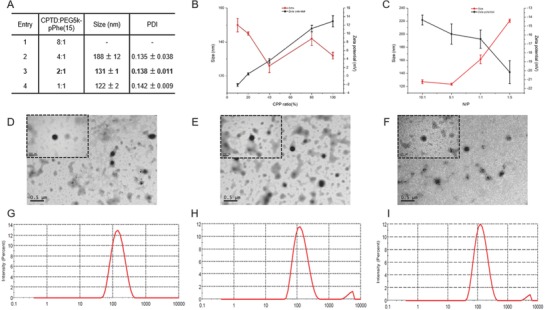
A) Formulation different weight ratio of CPTD with PEG_5k_‐pPhe(15) via nanoprecipitation. B) Size distribution of formulation 3 with different CPP modification. C) Size distribution and zeta potential of different N/P ratio of 40% CPP modification. TEM images of D) CPTD NPs, E) CPP‐CPTD NPs, and F) Apt/CPP‐CPTD NPs. Size distribution of G) CPTD NPs, H) CPP‐CPTD NPs, and I) Apt/CPP‐CPTD NPs measured by DLS.

Our novel sequentially triggered Apt/CPP‐CPTD NPs were prepared through a multistep approach as described in **Scheme** **1**. To achieve the tumor intracellular redox responsiveness, we developed a redox‐responsive dimeric CPTD prodrug based NPs according to our previous work.[[qv: 18b]] Such NPs comprised of a redox‐responsive dimeric CPTD prodrug and amphiphilic copolymer as a stabilizer. Compared to prototype CPT, a rigid molecule structure, which could easily form crystals/aggregates during drug formulation, our less‐rigid dimeric CPTD prodrug with freely rotatable bonds and a phenol ring could prevent the formation of long‐distance‐order structure and large aggregates during drug formulation, resulting in high‐drug loading NPs. Besides, the disulfide side chain in the CPTD prodrug enables the tumor intracellular intelligent redox‐responsive drug release via self‐cyclization and 1,4‐elimination reaction.[[qv: 18b,19]] The amphiphilic copolymers, mPEG_5k_‐pPhe(15) and N_3_‐PEG_5k_‐pPhe(15), were synthesized via ROP reaction in the presence of mPEG_5k_‐NH_2_ and N_3_‐PEG_5k_‐NH_2_ as the initiator, and l‐phenylalanine *N*‐carboxyanhydride (Phe‐NCA) as the monomer. The characterization peaks of PEG at 3.54 ppm (methylene groups), Phe at 4.5 ppm (methylene of benzyl group, EG) and at 7.21 ppm (phenolic group) were found in ^1^H‐NMR spectra shown in Figure S1 of the Supporting Information. The molar composition ratio of EG to Phe was 113:15 and conversion of monomer Phe to polymeric pPhe was 91%, calculated by ^1^H‐NMR (Figure S1, Supporting Information). The amphiphilic copolymer acted as surface stabilizer to further encapsulate the dimeric CPTD prodrug and formed the stable nanoparticle CPTD NPs via nanoprecipitation (Scheme 1). By modulating weight ratio of CPTD and amphiphilic copolymer mPEG_5k_‐pPhe(15), we prepared a series of CPTD prodrug NPs with different particle sizes and stability (**Figure** **1**A). Considering the suitable size for EPR effect^20^ and higher drug loading efficacy for drug delivery, we optimized the weight ratio of CPTD to mPEG_5k_‐pPhe(15) as 2:1, which showed the most uniform NPs with average particle size of 131 ± 1 nm and polydispersity index of 0.138 ± 0.011 (Figure 1G).

To enable the NPs with PDAC penetration ability, we then introduced CPP peptide onto CPTD NPs, since CPP peptide has performed excellent therapeutic agents permeability after intratumoral injection in PDAC.[[qv: 8a]] The azide group at the end of N_3_‐PEG_5k_‐pPhe(15) terminus could react with 5‐hexynoicacid CPP peptide (CPP) in the presence of Cu(I) catalyst to form CPP‐PEG_5k_‐pPhe(15). By adjusting the weight ratio of mPEG_5k_‐pPhe(15) and CPP‐PEG_5k_‐pPhe(15), we prepared a serious of CPP‐modified CPTD NPs. As shown in Figure 1B, because of the positively charge CPP, the zeta potential of CPP‐modified CPTD NPs increased from −2.1 ± 0.4 mV to 12.9 ± 1.3 mV with the CPP‐PEG_5k_‐pPhe(15) ranging from 0% to 100%. Considering the stability and optimized size for PDAC penetration, we selected a 40% CPP modification, which showed relatively small size of 126 ± 4 nm and slightly positive zeta potential of for further use (Figure 1B,H).

Although CPP peptide performed excellent therapeutic agents permeability after intratumoral injection.[[qv: 8a]] Unfortunately, due to the lack of selectivity for target cells, CPP has shown excellent in vitro cell internalization yet undesired tumor accumulation in vivo.[[qv: 8a]] To avoid the off‐target accumulation of CPP‐CPTD NPs in vivo, we further utilized tenascin‐C targeting aptamer GBI‐10 as negatively charged camouflage for CPP shielding and also tumor‐homing property. As shown in Figure C of the Supporting Information, we formulated the N/P of CPP and GBI‐10 from 10:1 to 1:5. With the negatively charged GBI‐10 aptamer addition, the zeta potential of Apt/CPP‐CPTD NPs shifted from −14.3 ± 0.6 mV to −20.2 ± 1.3 mV. The particular size increased greatly from 126 ± 4 nm to 221 ± 2 nm, also due to the addition of negatively charge aptamer. Considering the suitable size and particle charge for drug delivery, we optimized N/P as 5:1 with a size of 124 ± 2 nm and zeta potential as −16.5 ± 1.0 mV (Figure 1C,I). The drastic zeta reversion from positive charge to negative charge indicated the successful camouflage of GBI‐10 aptamer onto CPP‐CPTD NPs. CPTD NPs, CPP‐CPTD NPs, and Apt/CPP‐CPTD NPs all revealed uniform spherical morphology observed by TEM (Figure 1D–F). As shown in TEM images, one unique phenomenon was the extremely condensed drug core, which indicated the high drug loading of our dimeric NPs. To validate the high drug loading ability, we also measured the CPT loading rate in Apt/CPP‐CPTD NPs by HPLC, which was 26.2% ± 1.6%, much higher than conventional polymeric micelles or inorganic NPs.^21^ Such high drug loading was achieved due to the dimeric structure of CPTD prodrug, which included freely rotatable bonds and a phenol ring. Such structure was much less rigid comparing to prototype CPT, which could effectively prevent large aggregates/crystals during drug formulation, loading more prodrug into NPs with higher drug loading rate.

### 2.2 Redox Responsive CPT Release Profile of Apt/CPP‐CPTD NPs In Vitro

**Figure 2:**
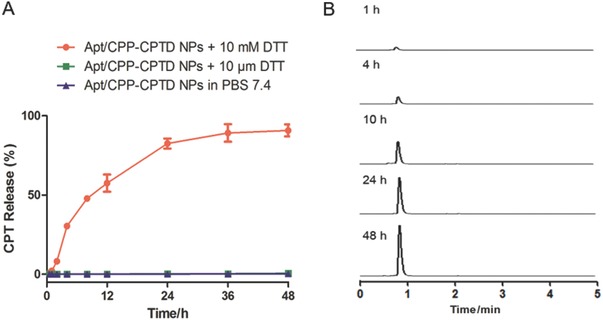
A) In Vitro CPT release from Apt/CPP‐CPTD NPs triggered by different concentrations of DTT in PBS 7.4 at 37 °C. Data were presented as means ± SD (*n* = 3). B) HPLC analysis of CPT release from Apt/CPP‐CPTD NPs in the presence of 10 × 10^−3^ m DTT as thiol trigger.

To verify the controllable tumor microenvironment responsive drug release property, we investigated the in vitro drug release behavior of Apt/CPP‐CPTD NPs in PBS 7.4 buffer with different dithiothreitol (DTT) concentration (10 × 10^−3^ m and 10 × 10^−6^ m) mimicking the intracellular tumor redox microenvironment. As shown in **Figure** **2**, negligible cumulative CPT release of Apt/CPP‐CPTD NPs was observed at pH 7.4 with 10 × 10^−6^ m DTT, mimicking the physiological redox potential. However, with the presence of 10 × 10^−3^ m DTT, representing the intracellular redox potential, as high as 90.8% ± 3.8% of CPT was released over 48 h from Apt/CPP‐CPTD NPs. Such huge difference indicated the successful tumor intracellular redox controlled drug release of Apt/CPP‐CPTD NPs. To be more specific, we also fitted the release curve of Apt/CPP‐CPTD in 10 × 10^−3^ m DTT with first order dynamic fitting equation. The fitting equation was *y* = 114.8 * (1 – exp (−0.0468*x)). And the release rate constant was 0.0468.

The sensitive redox controlled drug release of Apt/CPP‐CPTD NPs relied on the unique chemical structure of dimeric CPTD prodrug. As shown in Scheme S1 of the Supporting Information, two CPT molecules were deliberately conjugated to 2,6‐bis(hydroxymethyl)‐aniline through carbonate linkage. The amine group of aniline was protected by the disulfide bond. Meanwhile, the dimeric prodrug was encapsulated and protected in the inner core of Apt/CPP‐CPTD NPs. Encountered with high redox potential in intracellular compartment, the disulfide bond in CPTD side chain could be cleaved through the thiol‐disulfide exchange. The exposed thiol group further cyclized toward the carbonyl group and released the arylamine in the CPTD structure. The exposed amine group of aniline could induce electronic transferation and go through a self‐elimination reaction to release prototype CPT from the prodrug (Scheme S1, Supporting Information).

### 2.3 Investigation of Cellular Uptake and Internalization Mechanisms in PADC Cells

The efficient cellular uptake is prerequisite for efficient drug delivery. Many factors, such as particle size, zeta potential, ligand–receptor interaction have great effect on cellular uptake.^22^ CPP, which contains positively charged amino acid residues, is capable to translocating various nanomedicines across the cell membranes.^23^ To evaluate the sufficient internalization efficiency of our nanoparticle system, coumarin‐6 was encapsulated into different formulations for the in vitro nanoparticles tracing. To be specific, Miapaca cells were incubated with different prodrug nanoparticles, CPTD NPs, CPP‐CPTD NPs, and Apt/CPP‐CPTD NPs. Compared with CPTD NPs and Apt/CPP‐CPTD NPs, CPP‐CPTD NPs showed significantly enhanced cellular accumulation due to the cell‐penetration ability of CPP peptides (Figure S3, Supporting Information). It is understandable that Apt/CPP‐CPTD NPs showed less accumulation than CPP‐CPTD NPs in 2D cell level, since the positively charged CPP were camouflaged by GBI‐10 aptamer, resulting in less electrostatic interactions between Apt/CPP‐CPTD NPs and tumor cell membranes. Also compared with non‐carcinoma 293 cell, which expresses no tenascin‐C protein, Apt/CPP‐CPTD NPs showed higher cellular uptake in tenascin‐C excreting Miapaca cells.

To further elucidate the internalization mechanism of Apt/CPP‐CPTD NPs, several inhibitors were pretreated onto Miapaca cells to block several endocytosis pathways. Filipin (blocking caveolae‐mediated pathway), PhAsO (blocking clathrin‐dependent pathway), and colchicine (blocking micropinocytosis pathway) were applied as inhibitors to pretreat Miapaca while incubated with Apt/CPP‐CPTD NPs. As shown in Figure S2 of the Supporting Information, fluorescent signals of NPs and flow cytometry (Figure S3, Supporting Information) results demonstrated that the internalization of NPs was inhibited by filipin. Additionally, low temperature remarkably inhibited the cellular uptake as well. Therefore, CPP‐CPTD NPs were mostly internalized into Miapaca cells via caveolae‐mediated endocytosis, in accordance with previous studies.^24^

### 2.4 In Vitro Antitumor Efficacy in PADC Cells

**Figure 3:**
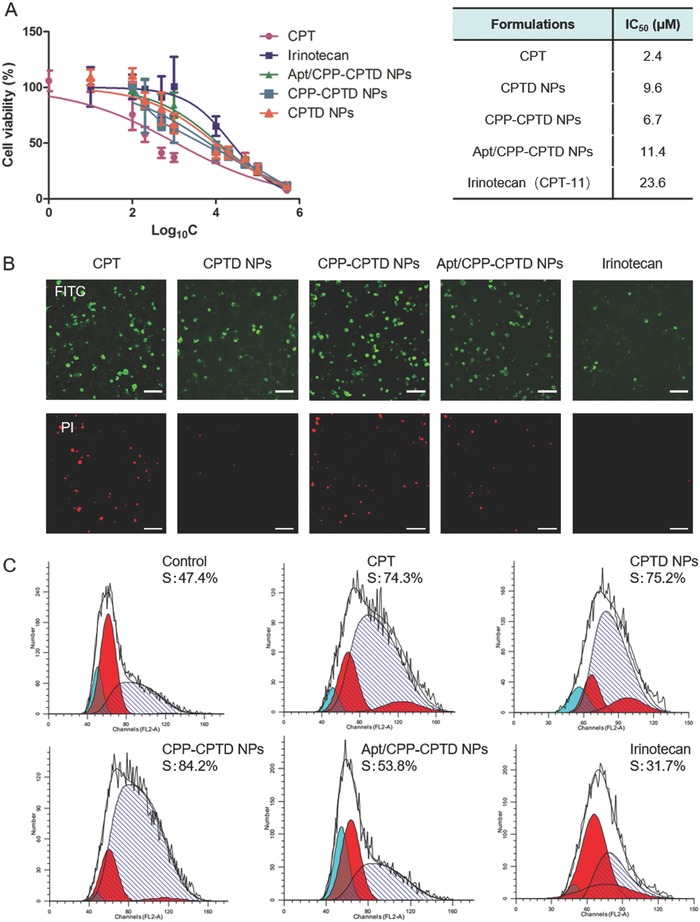
A) Cytotoxicity of CPT, CPTD NPs, CPP‐CPTD NPs, Apt/CPP‐CPTD NPs, and Irinotecan against Miapaca cells. B) Cellular apoptosis of Miapaca induced by CPT, CPTD NPs, CPP‐CPTD NPs, Apt/CPP‐CPTD NPs, and Irinotecan were investigated by fluorescence microscopy. Green: Annexin V‐FITC labeled apoptotic cells. Red: PI labeled dead cells. Scale bars represent 100 µm. C) Cell cycle distribution induced by CPT, CPTD NPs, CPP‐CPTD NPs, Apt/CPP‐CPTD NPs, and Irinotecan.

To evaluate the in vitro anticancer efficacy of NPs, MTT cytotoxicity assay on pancreatic cancer Miapaca cells and cellular apoptosis assays were implemented (**Figure** **3**). By incubating Miapaca cells with CPT solution, CPTD NPs, CPP‐CPTD NPs, Apt/CPP‐CPTD NPs, and commercially available drug irinotecan, significant cell proliferation inhibition was observed in a concentration‐dependent manner (Figure 3A). It was noticed that CPP‐CPTD NPs showed lower IC_50_ value (6.7 × 10^−6^ m) than that of CPTD NPs (9.6 × 10^−6^ m). Such difference is mainly due to the cell penetration property of CPP peptide modified onto the NPs. The IC_50_ of Apt/CPP‐CPTD NPs is slightly higher, which is understandable since the CPP peptide was camouflaged by negatively charged GBI‐10 aptamer. All the nanoparticle formulations showed slightly higher IC_50_ than prototype CPT solutions. Such phenomenon might be deduced to the relatively slower profile of the CPTD prodrug compared to the burst release of CPT molecules. All the nanoparticle formulations showed much higher cytotoxicity than irinotecan, a current clinically used CPT derivative. Previous reports have shown that CPT could trigger cell cycle arrest on S phase. We studied cell cycle arrest induced by CPT, CPTD NPs, CPP‐CPTD NPs, Apt/CPP‐CPTD NPs, and irinotecan. As shown in Figure 3C, all the formulations showed significant S phase arrest, indicating that intracellular thiols could successfully cleave the disulfide bond within NPs, releasing free CPT to induce cytotoxicity as designed. The results of cell cycle distribution shared consistency with the MTT study (Figure 3A).

To further elucidate the antitumor efficacy of NPs, we also performed the in vitro apoptosis assay on Miapaca cells by Annexin V‐FITC and PI assay (Figure 3B). Annexin V, which could specifically affinitive to calcium‐dependent phosphatidylserine binding protein, was used to indicate an early apoptosis (green). PI could specifically bind to intracellular DNA/RNA through damaged cell membrane, which indicated late apoptosis or necrotic cells (red). The results of apoptosis experiment shared consistency with the MTT study (Figure 3A), suggesting the potential of Apt/CPP‐CPTD NPs as a novel drug delivery system.

### 2.5 3D Miapaca Pancreatic Tumor Spheroid Penetration

**Figure 4:**
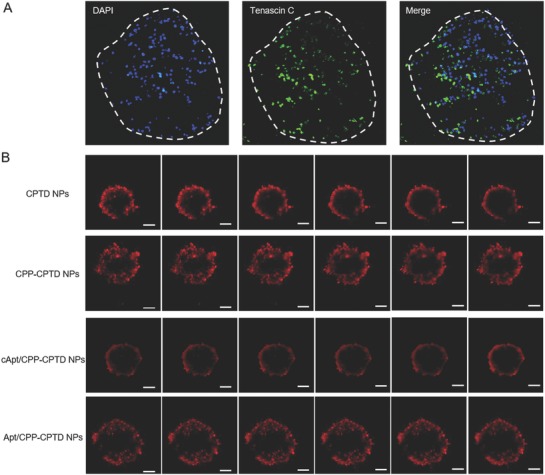
A) In vitro CLSM images of Miapaca 3D tumor spheroid, magnification scale 100×. Blue: DAPI stained nuclei, green: tenascin C. B) CLSM images showing in vitro penetration of fluorescence‐labeled CPTD NPs, CPP‐CPTD NPs, cApt/CPP‐CPTD NPs, and Apt/CPP‐CPTD NPs in Miapaca multicellular spheroids (MCSs). The MCSs were incubated for 4 h and measured by CLSM Z‐stack scanning with a 20 µm interval between consecutive slides. Scale bars represent 100 µm.

The dense tumor stroma in PDAC extracellular matrix causes the poor penetration profile of chemotherapeutics, greatly compromises the chemotherapy. As demonstrated, tumor spheroids are versatile 3D models for studying tumor biology due to their similarity in morphology and biological microenvironment to solid tumors.^25^ Therefore, we constructed a 3D tumor spheroid model to mimic the pathological penetration barrier of NPs in PDAC.[[qv: 25c]] As confirmed in **Figure** **4**A, high expression of tenascin‐C was found in Miapaca pancreatic tumor spheroids, which was excreted from PDAC cells into the ECM of tumor spheroids.

To further confirm our sequentially responsive nanoparticle with ECM‐triggered tumor penetration strategy, BODIPY‐labeled NPs were treated into PDAC 3D tumor spheroid for 4 h to assess the penetration capability. We chose CPTD NPs, CPP‐CPTD NPs, and control aptamer camouflaged NPs cApt/CPP‐CPTD NPs as controls. As shown in Figure 4B, CLSM Z‐stack scanning showed that CPTD NPs was mostly located on the periphery of tumor spheroids and dropped considerably in the interior areas. In comparison, the penetration capability of CPP‐CPTD NPs improved significantly compared with CPTD NPs due to cell penetrating property of CPP peptide. Such phenomenon confirmed the active penetration ability of CPP peptide. As for Apt/CPTD NPs and cApt/CPTD NPs, both formulations possessed similar physicochemical properties, yet showed significantly different penetration behavior in 3D tumor spheroids. Apt/CPTD NPs could penetrate to the centric position of tumor spheroids, while cApt/CPTD was constrained in the peripheral sections of tumor spheroid. Such phenomenon might due to the different bioeffect of random sequence aptamer (cApt) and tenascin‐C affinity aptamer GBI‐10. Because of the camouflaged CPP with negatively charged random sequence aptamer, (cApt)cApt/CPP‐CPTD NPs could not actively penetrate into 3D tumor spheroids. In comparison, due to the high affinity of GBI‐10 with tenascin‐C in the matrix of tumor spheroid, GBI‐10 could be detached from Apt/CPP‐CPTD NPs and the exposed CPP in Apt/CPP‐CPTD NPs could induce the deep penetration. These in vitro results suggest that our Apt/CPP‐CPTD NPs could perform ECM‐triggered penetration behavior, thus probably advantageous for in vivo tumor penetration capability.

### 2.6 In Vivo Tumor Accumulation and Tumor Penetration

**Figure 5:**
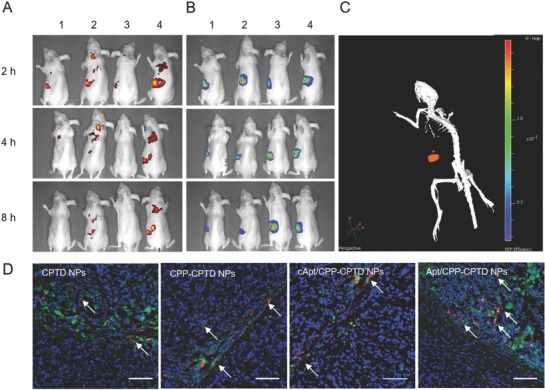
A) In vivo 2D fluorescent images of CPTD NPs(1), CPP‐CPTD NPs(2), cAPT/CPP‐CPTD NPs(3), and APT/CPP‐CPTD NPs(4) after i.v. administration at 2, 4, and 8 h. B) In vivo 2D bio‐luciferase signal images of CPTD NPs(1), CPP‐CPTD NPs(2), cAPT/CPP‐CPTD NPs(3), and APT/CPP‐CPTD NPs(4) after i.v. administration at 2, 4, and 8 h. C) 3D fluorescent image of APT/CPP‐CPTD NPs(4). D) Confocal images of tumor tissues after administration of CPTD NPs, CPP‐CPTD NPs, cAPT/CPP‐CPTD NPs, and APT/CPP‐CPTD NPs. Blue: DAPI stained nuclei, green: CD‐34, red: BODIPY‐labeled NPs. Scale bars represent 100 µm.

The in vivo tumor accumulation and tumor penetration efficacy of BODIPY‐labeled‐NPs on PDAC bearing nude mice was determined by near‐infrared imaging noninvasively. Giving that xenograft PDAC model was a kind of deep in situ tumor. We utilized bioluminescent luci‐Miapaca cells, which could metabolize d‐luciferin potassium and induce bioluminescence for tumor visualization. PDAC bearing nude mice were pretreated with d‐luciferin potassium to locate the PDAC tumor area (**Figure** **5**B). PDAC bearing nude mice were intravenously injected with CPTD NPs (1), CPP‐CPTD NPs (2), cApt/CPP‐CPTD NPs (3), and Apt/CPP‐CPTD NPs (4), respectively. As shown in Figure 5A, a stronger fluorescence signal at the PDAC site could be observed from 2 to 8 h after intravenously injected with CPP‐CPTD NPs and Apt/CPP‐CPTD NPs, compared with that of those treated with CPTD NPs and cApt/CP‐CPTD NPs. Such phenomenon might be due to the lack of actively targeting effect of CPP peptide in CPTD NPs or cApt/CPP‐CPTD NPs. Meanwhile, when compared with Apt/CPP‐CPTD NPs with that of CPP‐CPTD NPs, much less fluorescence signal was detected in mice treated with CPP‐CPTD NPs. During circulation, CPP‐CPTD NPs could perform active targeting and accumulate into tumor tissue via CPP peptide to some extent, however such positively charged NPs could be recognized and cleared by RES system. On the other hand, Apt/CPP‐CPTD NPs might escape from RES system due to Apt camouflage, and also accumulate into PDAC site via GBI‐10/tenascin‐C interactions. The affinity of GBI‐10 and tenascin‐C could lead to the exposure of CPP peptide, which induced further penetration and accumulation of NPs in the tumor site.

To further investigate the penetration efficacy of different NPs, neovascular and NPs were labeled with specific antibody and BODIPY, respectively (Figure 5D). As the rapid proliferation of tumor cells, the nuclei were rich and became denser than normal tissues, we chose the tumor sections and observed them by confocal laser scanning. Consistent with Figure 5A, Apt/CPP‐CPTD NPs showed the most accumulation in tumor tissues comparing to the other three groups. Most NPs were retained in neovascular, yet Apt/CPP‐CPTD NPs possessed most long‐distance extravasation. These in vivo results suggest the potential benefits of Apt/CPP‐CPTD NPs in PDAC treatment.

### 2.7 In Vivo Antitumor Efficacy

**Figure 6:**
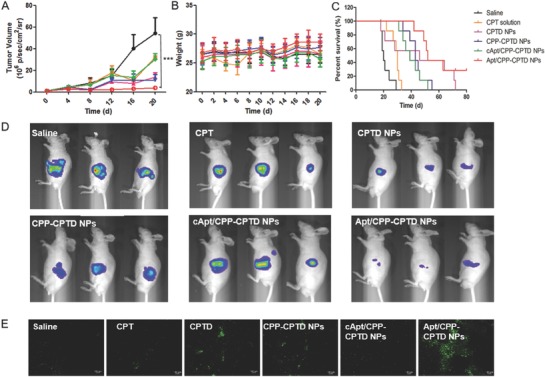
Antitumor efficacy of CPT solution, CPTD NPs, CPP‐CPTD NPs, cApt/CPP‐CPTD NPs, and Apt/CPP‐CPTD NPs on Miapaca xenograft mice. A) Tumor volume changes and B) body weight after i.v. injections of CPT solution, CPTD NPs, CPP‐CPTD NPs, cApt/CPP‐CPTD NPs, and Apt/CPP‐CPTD NPs with equivalent 10 mg kg^−1^ CPT on day 0, 3, 6, 9, and 12 (saline served as control). C) Survival curves were recorded after five doses of administration. D) Bioluminescence images of Miapaca xenograft models on day 12. E) Representative histological images of Miapaca tumor xenografts of treated groups using the TUNEL assay. Green: apoptosis cells.

To evaluate the antitumor efficacy of different NPs in vivo, PDAC bearing nude mice models were applied. The tumor volumes were calculated by the luminescence signals every 4 d (**Figure** **6**A). The mice body weight were recorded every 2 d to evaluate the general toxicity (Figure 6B). The survival rate of PDAC mice treated with different formulations was also recorded (Figure 6C). Apt/CPP‐CPTD NPs exhibited remarkably smaller tumor volume compared to the others (Figure 6A,D). Various degree of limited inhibition was found in the free CPT, CPTD NPs, CPP‐CPTD NPs, and cApt/CPP‐CPTD NPs treated groups due to a rapid clearance, lack of targeting ligand or inefficient CPP exposure. All the NPs‐treated groups maintained healthy weight and according to H&E staining results (Figure S4, Supporting Information), all formulations showed no significant systemic toxicity.

After the five doses course of therapy, the mice were then sacrificed and the tumor tissues were collected to carry out the TUNEL assay for detection of apoptosis (Figure 6E). The green staining signals of FITC labeled dUTP stained area indicated apoptosis site in tumor as they positioned the extensive DNA degradation. Samples from Apt/CPP‐CPTD NPs showed the most extensive apoptotic cells, which was consistent with previous tumor volumes results (Figure 6A).

We further investigated the collagen expression in PDAC tissues after five doses of treatment to evaluate if the tumor microenvironment, especially the ECM component, was affected by different treatment. To be specific, we imaged the collagen I expression in whole tumor sections (Figure S5, Supporting Information). Comparing with tumor section of control group, cApt/CPP‐CPTD NPs expressed decreased level of collagen I, especially in the marginal area. Such phenomenon might due to the unsuccessful detachment of cApt, cApt/CPP‐CPTD NPs was confined in the border area of tumor, and possessed marginal elimination of Collagen I. Meanwhile, Apt/CPP‐CPTD NPs could interact with tenascin‐C in ECM and detach GBI‐10 aptamer from CPP to restore the deep penetration behavior of CPP peptide. Therefore, Apt/CPP‐CPTD NPs could penetrate into the deep center of PDAC tumor while do not change the whole expression of collagen I. Such ECM‐triggered penetration strategy without harming the ECM environment could provide a new PDAC therapeutic strategy with reducing risk of tumor cell evasion from the original site.

## 3 Conclusion

In summary, we have developed a sequentially responsive nanoparticle with ECM‐triggered tumor penetration and redox responsive drug release profile. The novel Apt/CPP‐CPTD NPs selectively accumulated in tumor site via EPR effect and performed increased tumor penetration ability by the exposed CPP, which was activated in the PDAC‐related ECM component tenascin C. After endocytosis, CPTD prodrug could be sequentially triggered by intravenous redox potential and provide controlled drug release. Enhanced drug distribution in tumor site and good antitumor efficacy were observed both in vitro and in vivo. In the light of these results, the unique sequentially triggered nanoparticle with tumor penetration and intelligent drug release could serve well as a promising strategy for PDAC treatment.

## 4 Experimental Section

*Amphiphilic Copolymer Synthesis and CPTD Prodrug Synthesis*: Phe‐NCA was synthesized according to Fuchs‐Farthing method by triphosgene in anhydrous THF solution for 4 h at 50 °C under nitrogen atmosphere. The product was precipitated by slowly added into the anhydrous hexane and purified by washing with cold anhydrous hexane for three times. The solution was recrystallized at −20 °C overnight. The NCA monomer was then filtered and dried under vacuum for the following use.

mPEG‐pPhe(15) and N_3_‐PEG‐pPhe(15) were synthesized via an ROP reaction according to previous work with minor change.[[qv: 16a,b]] Briefly, mPEG‐NH_2_ or N_3_‐PEG‐pPhe(15) (1 g, 0.2 mmol) was dissolved in anhydrous DMF (10 mL) and added with Phe‐NCA (612 mg, 3.2 mmol) at 50 °C under nitrogen protection. After 48 h, the copolymer mixtures were precipitated into cold diethyl ether and filtered. The white products were dried under vacuum. Modification of CPP peptide to N_3_‐PEG‐pPhe(15) was performed via click reaction in the presence of Cu (I) as catalyst. The copolymer N_3_‐PEG‐pPhe(15) (500 mg, 0.1 mmol) and excess CPP peptide (2 eq.) were dissolved in DMF (5 mL) under argon. CuI (0.5 eq.) and DIPEA (1 eq.) was added and the reaction was stirred at room‐temperature overnight. The product CPP‐PEG‐pPhe(15) was purified by dialysis against 10 × 10^−3^ m EDTA for 24 h, DI water for another 24 h, followed by freeze‐drying.

Redox responsive dimeric CPTD prodrug was synthesized according to previous procedure.[[qv: 18b]] All the intermediate compounds were confirmed according to literature.

*Nanoparticle Preparation*: Different formulations of CPTD NPs were prepared by nanoprecipitation. CPTD and mPEG‐pPhe(15) were first dissolved in DMF at designated weight ratio and the drug concentration was 2 or 10 mg mL^−1^ for in vitro and in vivo study, respectively. The above mixture solution was added dropwise into 2 mL DI water under mild stirring (600 rpm) using a magnetic bar and dialysis against DI water for 12 h to remove residual organic solvents. Different formulations of CPP‐CPTD NPs were prepared by first mixing different ratio of CPTD, mPEG‐pPhe(15), and CPP‐PEG‐pPhe(15) at designated weight ratio in DMF. The above mixture solution was then added dropwise into 2 mL DI water with mild stirring (600 rpm) and dialysis against DI water for 12 h to remove residual organic solvents. Formulations of Apt/CPP‐CPTD NPs were prepared by mixing positively charged CPP‐CPTD NPs (with 40%, 80%, and 100% CPP modifications) with GBI‐10 aptamer or control aptamer (cApt) at certain N/P molar ratios (10:1 to 1:5) for 30 s. Then the above solutions were dialysis against DI water to remove residual aptamer. The BODIPY or coumarin‐6 labeled NPs were prepared according to the procedure mentioned above with addition of 5 wt% fluorescent dye in the nanoprecipitation step. All of the prepared formulations were stored in 4 °C in dark before use.

*In Vitro CPT Release Study*: In vitro CPT release profile of Apt/CPP‐CPTD NPs under different external stimulations were measured through a dialysis method (*n* = 3). 400 µL of Apt/CPP‐CPTD NPs were placed into dialysis bags (MWCO: 3500) with both ends sealed and then submerged into a centrifuge tube containing 10 mL release medium (PBS 7.4, PBS 7.4 containing 10 × 10^−6^ m DTT or PBS 7.4 containing 10 × 10^−3^ m DTT) at 37 °C and shaken at 100 rpm. An aliquot of solution (0.2 mL) was withdrawn from the release medium at selected time points and replaced with 0.2 mL fresh release buffer. The CPT concentration was measured by HPLC.

*In Vitro Antitumor Efficacy Study*: In vitro antitumor efficacy study was evaluated by MTT assay (*n* = 4) and cell apoptosis assay. As for MTT assay, Miapaca cells were seeded in 96‐well plates at a density of 3 × 10^3^ cells per well and incubated at 37 °C for 12 h. The cells were treated with CPT solution, CPTD NPs, CPP‐CPTD NPs, Apt/CPP‐CPTD NPs, and irinotecan at various concentrations for 48 h. Afterward, the drug mediums were removed and cells were rinsed carefully with Hank's for three times. Subsequently, 100 µL MTT solution (0.5 mg mL^−1^) was added in each well and the cells were incubated at 37 °C for another 4 h. The solution was then removed and 150 µL DMSO was added to dissolve the formazan crystal. The cell viability was then calculated using microplate spectrophotometer (BioTek, Winooski, USA) at 570 nm. Cells without drug treatment were served as control.

As for the cell apoptosis detection, Miapaca cells were seeded in 24‐well plates at a density of 1 × 10^4^ cells per well and incubated at 37 °C for 24 h until a confluence of 80%. The cells were treated with CPT solution, CPTD NPs, CPP‐CPTD NPs, Apt/CPP‐CPTD NPs, and irinotecan at normalized CPT concentration of 2 × 10^−6^ m at 37 °C for 6 h. The drug solutions were then removed and cells were further incubated for 12 h. The cells were stained with cell apoptosis kit according to the protocol. The cells were observed under the fluorescence microscope (Leica, Wetzlar, Germany, FL1 channel for Annexin V‐FITC, FL3 channel for PI).

The progression of cell cycle was determined by flow cytometry via Cell‐cycle Analysis Kit. Miapaca cells were seeded in 12‐well plates at a density of 1 × 10^5^ cells per well. The cells were incubated with CPT solution, CPTD NPs, CPP‐CPTD NPs, Apt/CPP‐CPTD NPs, and irinotecan at normalized CPT concentration of 2 × 10^−6^ m at 37 °C for 12 h. The drug solutions were then removed and cells were further incubated for another 12 h. Then the cells were harvested and stained according to Cell‐cycle Analysis Kit protocol and detected by FACS flow cytometer. The percentage of cell cycle phases was analyzed using the software of Flowjo 6.0.

*Preparation of Miapaca Spheroids*: Miapaca pancreatic tumor spheroids were prepared using hanging drop technique as reported previously.[[qv: 25c]] Briefly, 20 µL drops of the 0.24% methylcellulose‐culture medium solution containing 8000 cells were pipetted onto the lid of round cell culture dishes and were inverted over dishes containing 10 mL Hank's to maintain the humidity of the cell mixture. Hanging drop cultures were incubated at 37 °C with 5% CO_2_ for one week. The resulting 3D tumor spheroids were harvested by pipetting 10 mL DMEM gently onto the lid and suspended the 3D tumor spheroid in the media. The tumor spheroids were then transferred to 24‐well plate for treatment.

*Immunofluorescence Analysis*: To evaluate the expression of tenascin‐C in 3D pancreatic tumor spheroids, the spheroids were embedded in paraffin and sliced in 5 µm. After deparaffinization, rehydration, the sections were treated with citrate buffer with microwave heating for 10 min for antigen repair. After cooling down to room temperature, the sections were washed with PBS and blocked with 10% goat serum at room temperature for 30 min. The slides were then incubated with rabbit antitenascin C antibody at 4 °C overnight. After washed with PBS for three times, the sections were treated with goat antimouse IgG (Alexa Fluor 488) for 2 h and DAPI for 15 min, respectively, then observed under confocal fluorescence microscope (Carl Zeiss LSM710, Wetzlar, Germany) by 63× oil immersion lens.

*Confocal Microscopy of Pancreatic Tumor Spheroids*: The pancreatic tumor spheroids were incubated with series of BODIPY‐labeled nanoparticles (CPTD NPs, CPP‐CPTD NPs, cApt/CPP‐CPTD NPs, and Apt/CPP‐CPTD NPs) at equal concentration of 400 µg mL^−1^ BODIPY for 4 h. After incubation, the spheroids were washed with ice‐cold Hank's for three times and fixed with 4% formaldehyde for 30 min. The NPs treated spheroids were then subjected to confocal microscopy for 3D analysis.

*Tumor Implantation*: Miapaca orthotopic pancreatic cancer xenograft models were established according to previous study.^26^ Briefly, luci‐Miapaca cells were harvested using 0.05% trypsin solution and resuspended as single‐cell suspensions in PBS at a concentration of 2 × 10^6^ per 100 µL. Nude mice were anesthetized with 80 µL of 2% pentobrabital sodium solution. Then nude mouse's abdominal cavity was opened by a 5–10 mm transverse incision on the left flank. The tail of pancreas was carefully exposed and 2 × 10^6^ of luci‐Miapaca cells were slowly injected into the junction of pancreas body and tail. The pancreas was then placed back into the abdominal cavity. After 10 d, the nude mice were injected with d‐luciferin potassium at a dose of 3 mg per mouse to observe the pancreatic tumor and used for in vivo studies. The animal experiments were carried out in accordance with guidelines evaluated and approved by Fudan University Institutional Animal Care and Use Committee (IACUC) and ethics committee. The accreditation number is 2016‐03‐MHYY‐WQB‐01.

*In Vivo Biodistribution Study and Tumor Penetration Evaluation*: In vivo imaging study of the NPs distribution was performed on Miapaca orthodox pancreatic cancer xenograft models. Nude mice were intravenously injected with BODIPY‐labeled CPTD NPs, CPP‐CPTD NPs, cAPT/CPP‐CPTD NPs, and Apt/CPP‐CPTD NPs at equivalent BODIPY dose of 0.5 mg kg^−1^. The mice were anesthetized and visualized (IVIS Spectrum imaging system, Caliper Perkin Elmer, Waltham, USA) at Ex/Em 650/665 nm at 2, 4, and 8 h postinjection. Pancreatic tumor‐bearing mice were also intraperitoneal injected with d‐luciferin potassium at a dose of 3 mg per mice to observe the pancreatic tumor location via luminescence signal of luciferase contained tumor cells.

At 8 h, the pancreatic tumor‐bearing mice were anesthetized and perfused with 4% paraformaldehyde and the tumor tissues were harvested and immersed in 4% paraformaldehyde for 24 h. Subsequently, the tumor tissues were dehydrated with 15% and 30% sucrose solution for 24 h gradually. The tumor tissues were then frozen in OCT embedding medium (Sakura, Torrance, CA, USA) at −80 °C and sectioned at 20 µm thickness (Leica, CM1900, Wetzlar, Germany). The frozen slides were costained with DAPI and immunofluorescence stained with anti‐CD34 and subjected to confocal microscopy analysis (Carl Zeiss LSM710, Wetzlar, Germany).

*In Vivo Antitumor Efficacy Study*: Forty‐two Miapaca orthodox pancreatic cancer xenograft mice were randomized into six groups (*n* = 7), and intravenously administered with CPT solution, CPTD NPs, CPP‐CPTD NPs, cAPT/CPP‐CPTD NPs, Apt/CPP‐CPTD NPs, and saline, respectively, in every three days at a dose of 10 mg kg^−1^ CPT. The body weight of mice was recorded every other day. The tumor volume was evaluated by the luminescence signals intensity of luciferase containing tumor cells. The survival times were recorded and calculated from day 0 since pancreatic cinoculation to the day of death. Tumors excised from the pancreatic model on day 22 were fixed in 4% paraformaldehyde for 24 h and dehydrated with 15% and 30% sucrose solution for 24 h gradually. The tumor tissues were then frozen in OCT embedding medium (Sakura, Torrance, CA, USA) at −80 °C and sectioned at 20 µm thickness. The as‐prepared samples were then stained with TUNEL before being observed by fluorescence microscope (Leica, Wetzlar, Germany). The frozen slides were also immunofluorescence stained with anticollagen I and subjected to confocal microscopy analysis (Carl Zeiss LSM710, Wetzlar, Germany).

*Statistical Analysis*: Analysis was performed using GraphPad Prism Software and the results were presented as means ± SD. Statistical comparisons among multiple groups were assessed by one‐way analysis of variance (ANOVA). Statistical significance was defined as *p* < 0.05.

## Conflict of Interest

The authors declare no conflict of interest.

## Supporting information

Supplementary
