# Supplementary material for: Sequentially Triggered Nanoparticles with Tumor Penetration and Intelligent Drug Release for Pancreatic Cancer Therapy
Source: Adv Sci (Weinh). 2018 Feb 26;5(5):1701070. doi: 10.1002/advs.201701070 (PMC5979633; doi:10.1002/advs.201701070)

Copyright WILEY-VCH Verlag GmbH & Co. KGaA, 69469 Weinheim, Germany, 2018.

ADVANCED SCIENCE
Open Access

# Supporting Information

for *Adv. Sci.,* DOI: 10.1002/advs.201701070

## Sequentially Triggered Nanoparticles with Tumor Penetration and Intelligent Drug Release for Pancreatic Cancer Therapy

*Xi He, Xinli Chen, Lisha Liu, Yu Zhang, Yifei Lu, Yujie Zhang, Qinjun Chen, Chunhui Ruan, Qin Guo, Chao Li, Tao Sun, and Chen Jiang**

Copyright WILEY-VCH Verlag GmbH & Co. KGaA, 69469 Weinheim, Germany, 2016.

# Supporting Information

**Sequentially triggered nanoparticles with tumor penetration and intelligent drug release for pancreatic cancer therapy**

*Xi He, Xinli Chen, Lisha Liu, Yu Zhang, Yifei Lu, Yujie Zhang, Qinjun Chen, Chunhui Ruan, Qin Guo, Chao Li, Tao Sun, Chen Jiang* *

***Materials:*** Anhydrous dimethylformamide (DMF), anhydrous dichloromethane (DCM) and anhydrous tetrahydrofuran (THF) were purchased from Acros Organics (New Jersey, USA). L-phenylalanine (Phe) and triphosgene were purchased from TCI (Tokyo, Japan). Dithiothreitol (DTT), trifluoroacetic acid (TFA), acetonitrile (ACN) were purchased from J&K (Shanghai, China). α-Methoxy-ω-amino-poly(ethylene glycol) (mPEG-NH2, Mw 5000), α-azide-ω-amino-poly(ethylene glycol) (N3-PEG-NH2, Mw 5000) were purchased from Jenkem Technology (Beijing, China). CPT, irinotecan and D-luciferin potassium were purchased from Meilun Biotech (Dalian, China). MTT (3-(4,5-dimethylthiazol-2-yl)-2,5-diphenyltetrazolium bromide), phenylarine oxide, colchicine and filipin were purchased from Sigma-Aldrich (St. Louis, USA). Coumrin-6, 4, 6-diamidino-2-phenylindole (DAPI) and BODIPY FL were purchased from ThermoFisher (Shanghai, China). All of the chemical reagents were used as received without further purification. CPP peptide (N-C sequence: 5-hexynoicacid-YGRKKRRQRRR) were synthesized by ChinaPeptides Co. Ltd (Shanghai, China). The GBI-10 aptamer (CCCAGAGGGAAGACTTTAGGTTCGGTTCACGTCC) and control aptamer (cApt, ATCTTGAGCTTACGGCCAAGCAGTTTCCGCGGAG) were synthesized by GenScript (Nanjing, China). One step TUNEL Apoptosis Detection Kit, Annexin V-FITC/PI Apoptosis Detection Kit and Cell-cycle Analysis Kit were purchased from KeyGEN BioTECH (Nanjing, China). Anti-Tenascin-C antibody, anti-CD34 antibody,

anti-β III tubulin antibody, anti-collagen I antibody, goat anti-mouse IgG (Alexa Fluor 488), goat anti-rabbit IgG (Alexa Fluor 594) were purchased from abcam (Cambridge, UK). Other reagents were purchased from Sinopharm Chemical Reagent (Shanghai, China).

Pancreatic cancer cell Miapaca cell line was kindly provided from Prof. Xiaolin Wang, Zhongshan Hospital, Fudan University, Shanghai. Miapaca were cultured in Dulbecco's Modified Eagle Medium (DMEM) containing 10% fetal bovine serum (FBS), 100 U/mL penicillin G, and 100 μg/mL streptomycin at 37 ℃ under 5% CO2 humidified atmosphere.

Balb-c nude mice (male, above 20 g) were purchased from Department of Experimental Animals, Fudan University and carefully maintained under SPF laboratory conditions. All animal experiments were carried out in accordance with guidelines evaluated and approved by Fudan University Institutional Animal Care and Use Committee (IACUC) and ethics committee. Orthotopic pancreatic cancer xenograft model was constructed according to previous study.

***Characterizations:*** Synthetic copolymers and intermediated prodrug compounds were characterized by $^1$H NMR spectra measured by NMR spectrometer (400 MHz, Bruker, USA) using d-chloroform, d-DMSO as solvents. Size, PDI and zeta-potential of the prepared NPs were measured by DLS (Malvern 3600, Worcestershire, UK). The morphology of NPs were performed using B-TEM (B-TEM, Tecnai G2 spirit Biotwin, FEI, Hillsboro, USA). CPT quantification was performed by high-performance liquid chromatography (HPLC) on an Agilent HPLC system with a reverse phase C18 column (250×4.6 mm, 5 μm). Gradient method was adopted using 0.1% TFA-$H_2O$ and ACN as mobile phase **(Figure S2)** at a flow rate of 1mL/min. The injected volume was 20 μL and fluorescence detector (Agilent 1260 VWD) was set to monitor at 369/442 nm. The calibration curve was calculated with good linear correlation ($R^2$ = 0.9997). All quantifications were performed in triplicates.

***Drug loading measurement:*** Freshly prepared nanoparticle solution was dissolved in DMF and incubated with 100 mM DTT for 4 h. The amount of CPT was measured by HPLC using standard calibration curve. The weight of Apt/CPP-CPTD NPs was collected via lyphilization. The drug loading rate (DL) was calculated as DL = w(CPT)/[w(drug conugates)+ w(polymer)].

***Cellular uptake and internalization mechanism study:*** Miapaca cells were seeded in 6-well plates (Corning, New York, USA) at a density of $1\times10^5$ cells per well and incubated at 37 □ in a humidified 5% $CO_2$ atmosphere for 24 h before reaching a confluence of 80-90%. The cells were then incubated with coumarin-labeled CPTD NPs, CPP-CPTD NPs and Apt/CPP-CPTD NPs at a normalized concentration. For the internalization mechanism study, 1 μg/mL colchicine, 0.3 μg/mL PhAsO and 1 μg/mL filipin were applied to the cells as endocytic inhibitors respectively and 4 □ condition was used as energy blockage. 293 cells with no tenascin C expression were used as the negative control. After 20 min pre-treatment, Apt/CPP-CPTD NPs were added and cells were incubated for another 1 h. Then the medium was removed and cells were washed three times with Hank's before observed by fluorescence microscope (Leica, Wetzlar, Germany). For flow cytometry analysis, the cells were digested and harvested before analyzed by flow cytometer (BD FACSAria II, San Jose, USA) at 488 nm excitation with $1\times10^4$ cells recorded for each assay. Cells without any treatment were used as control.

***Immunohistological staining study:*** To further evaluate the biosafety of NPs, Miapaca orthodox pancreatic cancer xenograft models of different groups were sacrificed on day 22 and the main organs were harvested and fixed in 4% paraformaldehyde and embedded in paraffin. Hematoxylin and eosin (H&E) staining were conducted by Guge Biological Tech. Co. Ltd. (Shanghai, China).

**Scheme S1.** Proposed redox-controlled release mechanism of CPTD

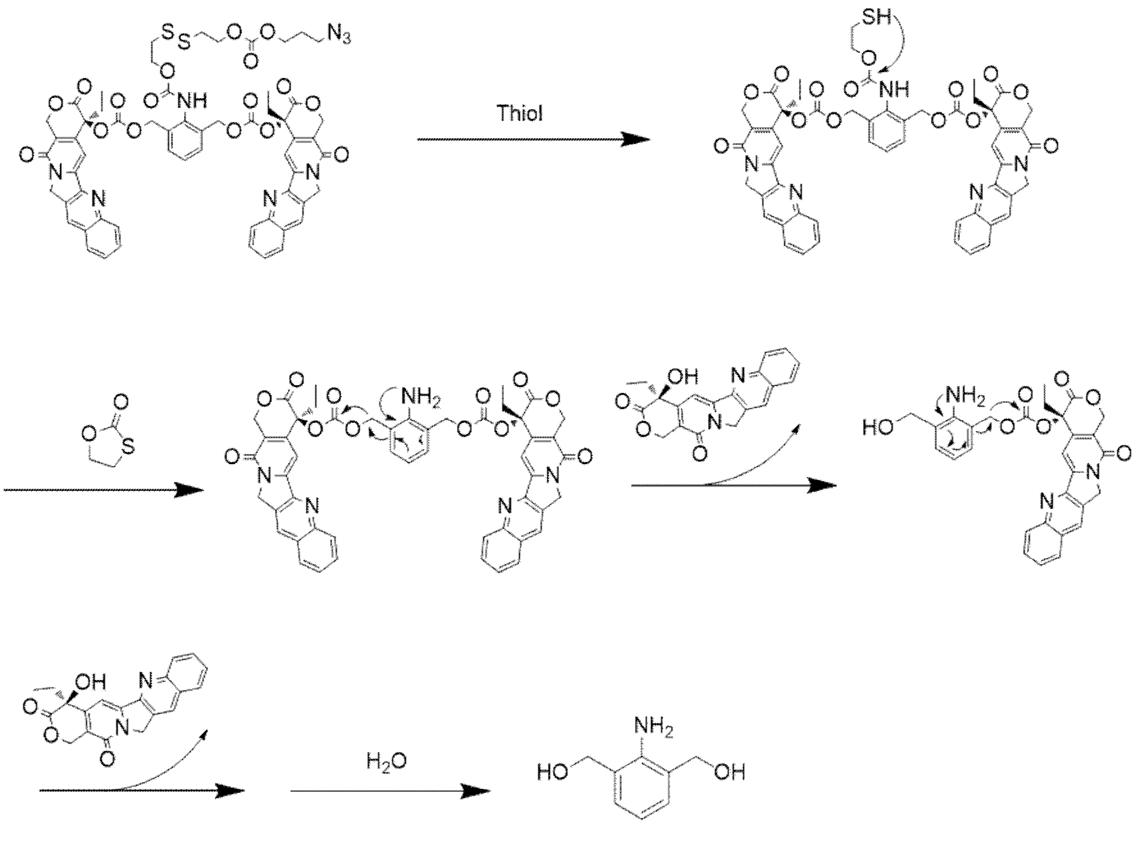


**Figure S1.** (A) $^1$H NMR of mPEG$_{5k}$-pPhe(15). (B) $^1$H NMR of N$_3$-PEG$_{5k}$-pPhe(15).

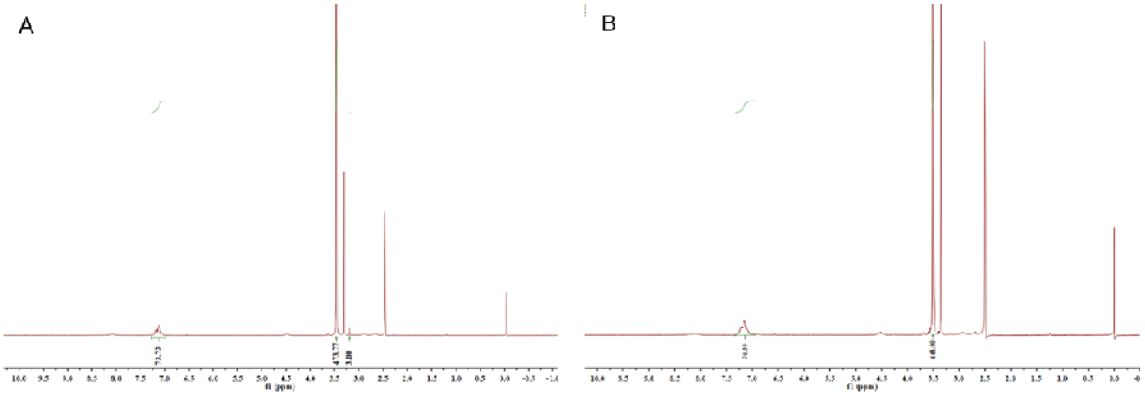

**Figure S2.** (A) HPLC method for CPT analysis. (B) HPLC Standard curve of CPT based on fluorescence ($\lambda_{em}$=369 nm, $\lambda_{ex}$=442 nm) ($R^2$=0.9997)

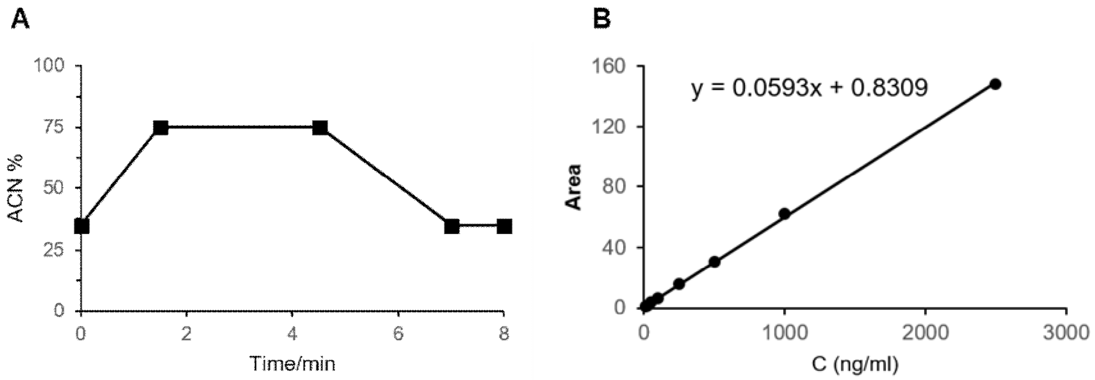


**Figure S3.** (A) Cellular uptake of CPTD NPs (a), CPP-CPTD NPs (b) and Apt/CPP-CPTD NPs after 30 min incubation. (d-h) Possible endocytosis pathway of CPP-CPTD NPs study. Cells were blocked by different inhibitors: 1 μg/mL filipin(d), 0.3 μg/mL PhAsO (e), 1 μg/mL Colchicine (f), cells were incubated at 4 □ (g). Cellular uptake of Apt/CPP-CPTD NPs in 293 cells with no tenascin C expression was used as negative control (h). (B) Quantitative results of cellular uptake and endocytosis pathway analyzed from flow cytometry analysis.

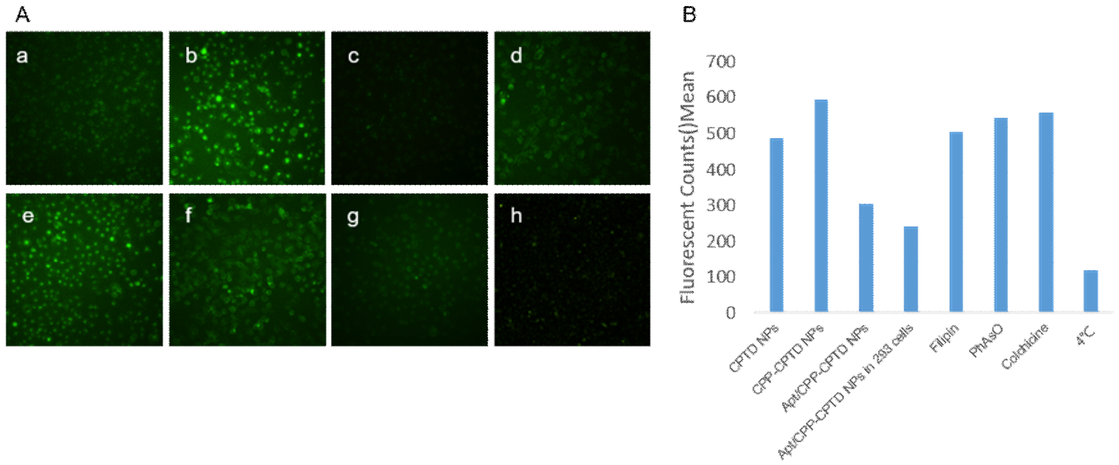

**Figure S4.** H&E staining of Saline(1), CPT(2), CPTD NPs(3), CPP-CPTD NPs(4), cAPT/CPP-CPTD NPs(5) and APT/CPP-CPTD NPs(6) after treatment

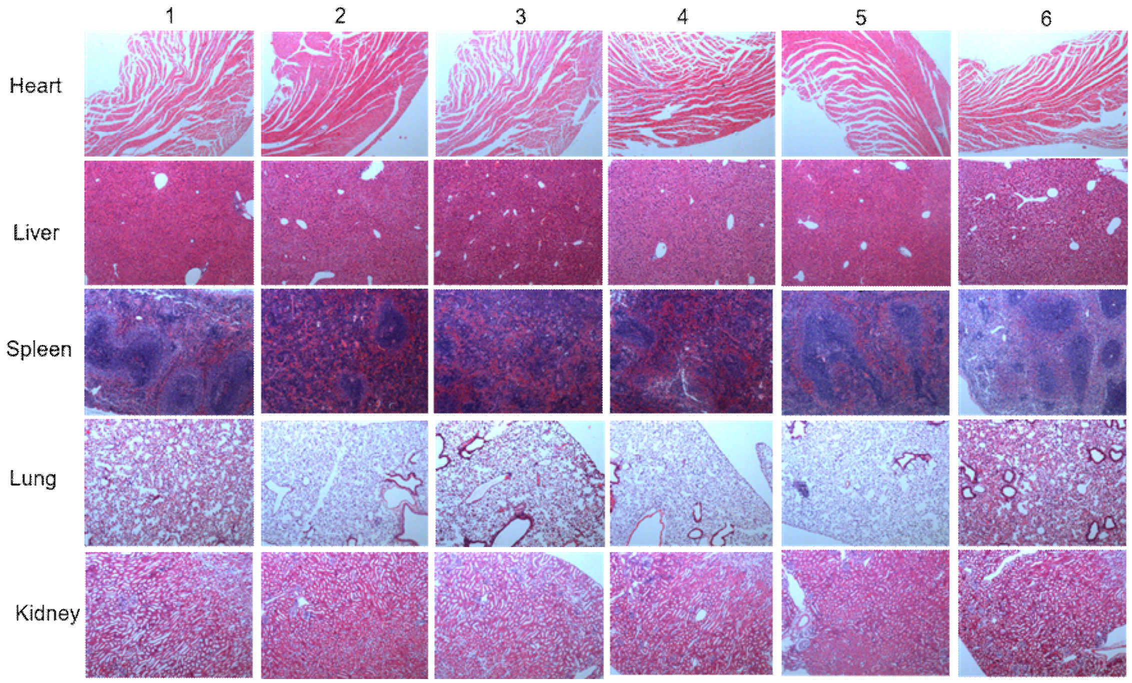


**Figure S5.** Collagen expression of whole tumor tissues sections after 5 doses of treatment. Green: collagen I, Blue: DAPI.

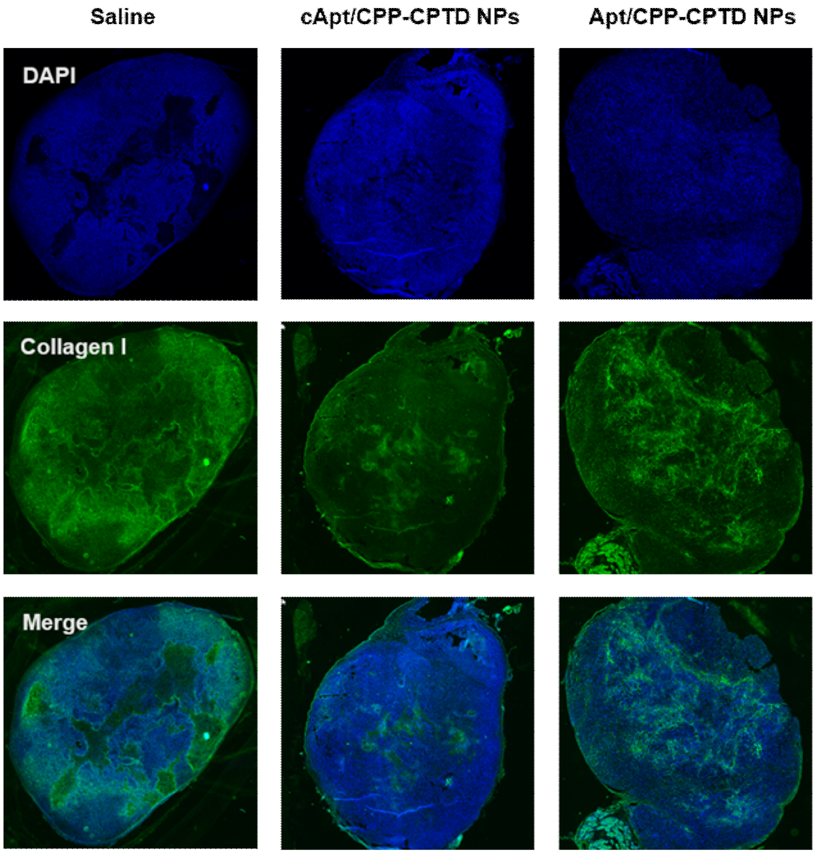

Supplement: Supplementary file 1 — Supplementary [file ADVS-5-1701070-s001.pdf]
